# The Chronic CARe for diAbeTes study (CARAT): a cluster randomized controlled trial

**DOI:** 10.1186/1475-2840-9-23

**Published:** 2010-06-15

**Authors:** Anja Frei, Corinne Chmiel, Hansueli Schläpfer, Beatrice Birnbaum, Ulrike Held, Johann Steurer, Thomas Rosemann

**Affiliations:** 1 Institute of General Practice and Health Services Research, University of Zurich, Zurich, Switzerland; 2 Horten Centre for Patient-oriented Research, University of Zurich, Zurich, Switzerland; 3 Division of Internal Medicine, University Hospital of Zurich, Switzerland; 4 Ärztenetz säntiMed, Herisau, Switzerland; 5 Schweizerischer Verband Medizinischer PraxisAssistentinnen, Bern, Switzerland

## Abstract

**Background:**

Diabetes is a major challenge for the health care system and especially for the primary care provider. The Chronic Care Model represents an evidence-based framework for the care for chronically ill. An increasing number of studies showed that implementing elements of the Chronic Care Model improves patient relevant outcomes and process parameters. However, most of these findings have been performed in settings different from the Swiss health care system which is dominated by single handed practices.

**Methods/Design:**

CARAT is a cluster randomized controlled trial with general practitioners as the unit of randomization (trial registration: ISRCTN05947538). The study challenges the hypothesis that implementing several elements of the Chronic Care Model via a specially trained practice nurse improves the HbA1c level of diabetes type II patients significantly after one year (primary outcome). Furthermore, we assume that the intervention increases the proportion of patients who achieve the recommended targets regarding blood pressure (<130/80), HbA1c (=<6.5%) and low-density lipoprotein-cholesterol (<2.6 mmol/l), increases patients' quality of life (SF-36) and several evidence-based quality indicators for diabetes care. These improvements in care will be experienced by the patients (PACIC-5A) as well as by the practice team (ACIC). According to the power calculation, 28 general practitioners will be randomized either to the intervention group or to the control group. Each general practitioner will include 12 patients suffering from diabetes type II. In the intervention group the general practitioner as well as the practice nurse will be trained to perform care for diabetes patients according to the Chronic Care Model in teamwork. In the control group no intervention will be applied at all and patients will be treated as usual. Measurements (pre-data-collection) will take place in months II-IV, starting in February 2010. Follow-up data will be collected after 1 year.

**Discussion:**

This study challenges the hypothesis that the Chronic Care Model can be easily implemented by a practice nurse focused approach. If our results will confirm this hypothesis the suggestion arises whether this approach should be implemented in other chronic diseases and multimorbid patients and how to redesign care in Switzerland.

## Background

Chronic diseases and multimorbidity are the major challenge for the future health care system [1]. Since chronic diseases not only impact individuals' quality of life but also represent a tremendous economical burden, many approaches have been made to increase patients' quality of life and to reveal affordable disease management tools. Based on the evidence of these interventions, summarized in a review of Tsai et al., Wagner and colleagues developed the Chronic Care Model (CCM) as a conceptual framework [2-5]. The aim of the CCM is to integrate all evidence-based concepts or approaches into this conceptual framework. One of the major problems addressed by the CCM is the fact that current care of chronically ills is often reactive and triggered by actual problems instead of being proactive, structured and planned [6]. Based on the current evidence, the CCM contains 6 key dimensions of care: Organization of health care, clinical information systems, delivery system design, decision support, self-management support, and community resources.

Most of the data supporting the concept of the CCM has been collected in settings in the U.S., primarily HMOs, which do not exist in most European countries. In Switzerland, the majority of the practices are single handed and privately owned. The system is very physician-centered; practice nurses are only marginally involved in the diagnostic and therapeutic process. One reason might be that the proportion between physicians and inhabitants is very high in Switzerland compared to other developed countries. As many other countries, Switzerland is faced with huge demographic changes, leading to a rising number of older and chronically ill inhabitants. On the other hand, the number of GPs is decreasing over many years. Consequently, new, more team-oriented approaches in the care for chronically ills are indispensable. Nevertheless, GPs in small, single handed practices are momentarily the main provider of care for chronically ills including diabetics in the Swiss health care system. Large specialist care centers do not exist; endocrinologists are only used for consultations in difficult treatment situations. Interestingly, electronic patient records, an important tool in the treatment and especially follow-up of chronically ills are not well established in Switzerland. As recent data showed, only about 10% of GPs work with electronic patient records (Zoller et al., Swiss Medical Weekly, submitted). Therefore, it is unclear if and to what extent the results generated in the U.S. settings are transferable to Europe or Switzerland in particular. Due to its widespread acceptance the CCM has achieved, an intense discussion has started among European physicians if and how the CCM or its components can be implemented in health care [7,8].

Regarding diabetes, valid data on the prevalence in Switzerland is not available. In Germany for example the overall prevalence of diabetes for the 25- to 65-year-old population was 5.34% in 2002-2005 [9] and therefore less than in the U.S. where the prevalence was estimated to 20.8 million (7% of the population) [10]. Data form the U.S. show that the expenditures for patients with diabetes are at a rate 2.3 times higher than those without the disease [11].

Several studies have shown that there is considerable potential to improve the process and outcome of diabetes care in general practice. Some studies showed that 28% diabetic patients in general practices have poor glycemic control and 55% have a high body mass index [12]. Structured programs as the disease management programs introduced in Germany have shown to improve the quality of care [13] but results regarding clinical outcomes are not yet available. Unfortunately, such programs involving the practice nurse in the treatment have not been assessed in Switzerland.

Implementing changes in primary care, especially new concepts of care with the complexity as the CCM, represents a big challenge. It is well known that strategies to implement changes in primary care vary widely in their effectiveness to change clinical practice. For instance, passive dissemination of information is generally ineffective for altering health professional behavior [14], while audit with feedback has a small to moderate effect [15]. Multifaceted interventions targeting different barriers to change tend to be more effective than single interventions [16]. Educational outreach visits can also change professionals' behavior [17]. Self management is the primary goal of diabetes education interventions. Diabetes associated complications are preventable in most cases. Research showed that control of glucose (HbA1c), blood pressure (BP) and low-density lipoprotein (LDL) cholesterol seem to be the most important targets to avoid microvascular and macrovascular complications of diabetes [18,19].

Diabetes impacts quality of life tremendously [20,21] and increases the incidence of depression [22], which is an important comorbidity in most chronic diseases. Depression or depressed mood has a negative effect on patients' ability to carry out self-management [23] and can in consequence worsen the glycemic control [23,24]. Prior studies in diabetes indicated that depression is recognized and treated in about 33% of patients, even though psychotherapy and psycho pharmacotherapy have been shown to have significant beneficial effects on mood and glycemic control [25-27].

In daily routine an obvious gap between recommendations and clinical practice can be observed, since the majority of patients (93%) do not achieve the recommended goals for HbA1c, LDL and BP [28-30]. The figures vary depending on the observed setting, but it can be assumed that over one-third of adults have HbA1c levels above recommended thresholds [29]. Other studies indicated that 32% are above a BP of 130/80mmHg, 66.2% have LDL values >2.6 mmol/l and nearly a third of the patients do not receive recommended annual eye (32.3%) or foot (31.7%) exams [31]. Regarding the thresholds in this study, we decided to choose a HbA1c level of 6.5% since the guidelines of the AHA and ADA recommend a level of 7.0% as general target and a level of 6.0% as ideal target [32].

This study challenges the hypothesis that implementing elements of the Chronic Care Model via a specially trained practice nurse in team work with the general practitioner improves the HbA1c level of diabetes type II patients in small, single handed practices in Switzerland significantly after one year (estimated change: 0.5) and increases the proportion of patients who achieve the recommended targets [32] regarding blood pressure (<130/80), HbA1c (=<6.5%) and LDL-cholesterol (<2.6 mmol/l) significantly.

Furthermore, we assume that this implementation improves patients' quality of life (SF-36) and several relevant quality indicators for diabetes care.

Finally we hypothesize that these improvements in care aiming at a better accordance with the CCM will be experienced by the patients as well as by the practice team, reflected in increased scores in the Patient Assessment of Chronic Illness Care (PACIC-5A) and Assessment of Chronic Illness Care (ACIC) instruments.

## Methods/Design

### Outcome-Parameter

The outcome parameters and instruments which are used in the study are summarized in Table 1.

**Table 1:** Outcome-parameters and instruments of the study

Outcome-Parameter (Patient)	Parameter/Instrument
**Primary Outcome**	HbA1c level

**Secondary outcomes**	

**- Guideline adherence (recommended treatment goals)**	% of patients reaching goal HbA1c (< 6.5%), LDL-Cholesterol (< 2.6 mmol/l); blood pressure (< 130/80mmHG)

**- Quality of life**	SF-36

**- Process quality**	

	% patients receiving at least one eye examination per year

	% patients receiving at least one food examination per year

	% patients receiving at least one nephropathy screening per year

	% patients receiving at least one neurological testing

**- Accordance to the CCM**	PACIC 5A (patients perspective)

	ACIC (provider perspective)

**Confounder control**	PHQ-9 (depression)

### Primary outcome

The primary outcome is the glycated haemoglobin (HbA1c) level. The power calculation is based on this parameter.

### Secondary outcomes

#### Guideline adherence (recommended treatment goals)

The proportion of patients who achieve the recommended targets for diabetes patients [32] regarding blood pressure (<130/80), HbA1c (=<6.5) and LDL-cholesterol (<2.6 mmol/l).

#### Quality of Life (QoL)

Even though the HbA1c is the most common parameter in diabetes trials, the quality of life is an important outcome parameter. Furthermore, recent studies have shown that decreasing the HbA1c aggressively can be associated with severe side effects, most likely due to hypoglycemia. In general, disease specific instruments should be favored against generic instruments, especially due to a higher sensitivity to change. Regarding diabetes, several instruments to assess QoL have been developed. Since none of them has so far proven to be superior over the well established SF-36 [33], which has already been used in large diabetes trials in primary care, we decided to use this instrument.

#### Quality indicators

Quality indicators can be based on process measures or clinical outcome measures. To enable international comparisons, we decided to asses quality indicators for diabetes according to the recommendations of the American Diabetes Association [34]. These quality indicators for diabetes care are also used e.g. by the British National Diabetes Quality Improvement Alliance. The indicators reflect the physical examination (incl. feet) and referrals (incl. eye):

P1: Percentage of patients receiving at least one nephropathy screening per year

P2: Percentage of patients receiving a dilated eye examination or evaluation of retinal photography by an ophthalmologist or optometrist per year

P3: Percentage of patients receiving at least one foot examination per year

P4: Percentage of patients receiving at least one peripheral neurological testing per year

#### Accordance to the Chronic Care Model (CCM)

##### Patients' perspective

Patients assessment of the provided care will be assessed with the Patient Assessment of Chronic Illness Care (PACIC 5A) which has been developed to assess congruency of provided health care to the CCM [35]. It is organized according to the key elements of the CCM and assesses the behavior of professionals and practice teams from a patient's perspective. The PACIC 5A contains 20 items assessing 5 scale constructs: patient activation, delivery system design/decision support, goal setting/tailoring, problem solving/contextual, follow-up/coordination. "Patient activation" assesses to what extent the patient was motivated and supported by the physician to initiate changes. "Decision support" assesses if the patient was supported e.g. by booklets and how satisfied he was with the organization of his care. "Tailoring" assesses to what extent general instructions and suggestions were adapted to his personal situation. "Problem solving" addresses how the physician dealt with problems which interfered with achieving predefined goals. Finally, "Follow-up" addresses how frequently and consequently the whole process was followed-up. Recently, a German version of the PACIC 5A has been validated in a sample of osteoarthritis patients [36]. The "5A" model represents an evidence-based approach to induce a behavioral change [37]. Glasgow et al. expanded the PACIC 5A by including 6 items assessing to what extent physicians' counseling reflects the 5A-approach which represents the recommended counseling approach for behavioral changes according to the recommendations of the US Preventive Services Task Force (USPSTF). The PACIC 5A was validated in a sample of diabetes patients [38].

##### Provider perspective

To assess accordance to the CCM of the health care provider's perspective, the Assessment of Chronic Illness Care (ACIC) [39] will be used. The ACIC is aimed at organizational teams to help identifying areas for improvement in their care for chronic illnesses and to evaluate the level and nature of improvements made in their system. It consists of 28 items covering the six areas of the CCM: Organization of the healthcare delivery system (6 items), community linkages (3 items), self-management support (4 items), decision support (4 items), delivery system design (6 items) and clinical information systems (5 items). Responses fall within four descriptive levels (D, C, B, A) of implementation ranging from D "little or none" to A, a "fully implemented" intervention. Within each of the four levels, respondents are asked to choose one of three ratings of the degree to which that description applies. The result is a 0-11 scale, with categories defined as follows: 0-2 (little or no support for chronic illness care), 3-5 (basic or intermediate support for chronic illness care), 6-8 (advanced support) and 9-11 (optimal, or comprehensive, integrated care for chronic illness). Subscale scores for the six areas are derived by summing the response. Bonomi et al. showed all six ACIC subscale scores to be responsive to health care quality-improvement efforts [39]. A translated and culturally adapted version into German (G-ACIC) has just been validated by the authors (submitted).

### Confounder control

Depression has been revealed as a potential confounder on quality of life and on satisfaction with care in most chronic diseases, including diabetes. Depression is also an independent predictor of mortality in many chronic diseases [40,41]. It also influences the assessment of the provided care with the PACIC 5A. Thus, depression will be assessed by means of the Patient Health Questionnaire, short form PHQ-9 [42]. This instrument has been proven to be a valid and reliable tool and has already been used in previous studies in primary care as e.g. the PraxArt trial [43,44].

### Study design

**Figure 1:**
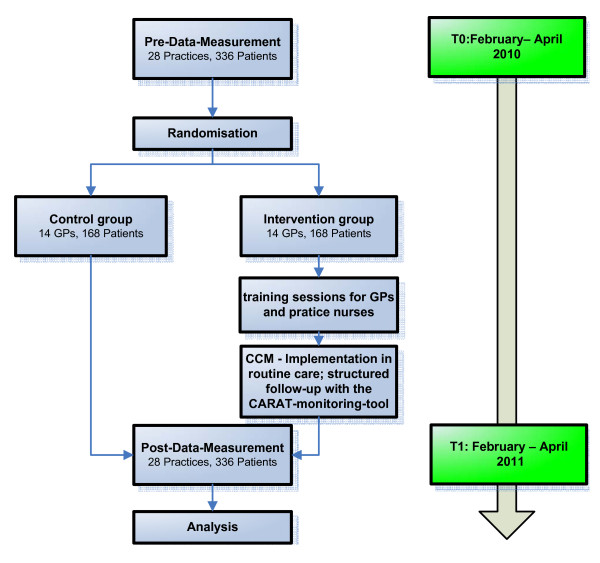
**Flow chart of the study**.

The study is a (prospective) cluster-randomized, open, two-armed intervention study with the GP as the unit of randomization. The design of a cluster randomized study was chosen because this design has optimal internal validity, while avoiding contamination of interventions associated with patient randomization. The flow chart of the study is described in Figure 1.

### Sample size

Due to its hierarchical structure, sample size calculations for cluster randomized trials differ from sample size calculations for common RCTs [45,46]. The required number of patients increases with the intracluster correlation, estimated in the intracluster correlation coefficient [47]. Based on our previous studies and on data available at the website of the University of Aberdeen [48], we assumed an ICC of 0.04 for the primary outcome. As primary outcome we defined the HbA1c. The sample size calculation was performed with the Cluster Randomization Sample Size Calculator ver.1.02 of the University of Aberdeen.

Unfortunately, no epidemiological data regarding the HbA1c is available from the Swiss primary care setting. Based on previous data and on our inclusion criteria (HbA1c > 7.0%) we assumed a mean HbA1c of 7.7% at pre-data-measurement time and aimed at a reduction in the HbA1c of 0.5% points with the intervention. In accordance to the data from the "Diabetes in Germany" (DIG)-study [49] and the ACCORD trial [50], the SD was assumed to be 1.2. The power was defined as 80%. The significance level was defined to be 0.05. Based on these assumptions and definitions we have to include 12 patients and 11 practices in each arm. In cluster randomized trials a higher drop out rate has to be assumed since the drop out of one cluster leads to the loss of all patients in a cluster. Therefore we assumed a drop out rate of 20%, resulting in 14 practices in each arm and 28 in total [45,46,51].

### Recruitment of GPs and randomization

The GPs are the unit of randomization. They are eligible for randomization if they participate in the routine primary care of unselected patients to assure that patients of all social levels have unlimited admission to the practice. If they are working in a non-single-handed practice, it is important that they have their own patients which can clearly be allocated to them. From an address database of all GPs in the area of Zurich, St. Gallen and Appenzell every second GP (about 800 GPs in total) was randomly selected, informed about the study and invited to an information meeting by a formal letter of the Department of General Practice and Health Services Research of the University of Zurich at the end of November 2009. Additionally, the project was presented in several quality circle meetings in doctors' networks (regions Zurich, Winterthur, St. Gallen). The aim of the information meeting was to provide the GPs with detailed information on the purpose and the associated effort for participating as well as to give an overview on the course of the whole study. If a GP was not able to attend the meeting but was interested in participation, he was invited to contact the Department of General Practice and Health Services Research and was delivered detailed information about the study by a project assistant.

The GPs who finally agree to participate will be alphabetically ordered in a list with numbers from 1 to 28 (no constraint will be imposed to cluster randomization). With the statistical program SPSS (version 18.0), 14 figures out of 28 will be randomly generated which represent the intervention group. The GPs with the corresponding numbers will be allocated to the randomly generated figures and to the intervention group, respectively. The randomization will be performed by an independent assistant who is not involved in the study and who is blind to the identity of the GPs. GPs will be informed about the group they have been randomized to after the inclusion of patients has been completed to avoid any bias in including patients.

### Patient inclusion criteria

Eligible patients are identified through the registry based on laboratory. To be eligible for inclusion patients have to be adult and diagnosed with diabetes type II according to the recommendations of the Swiss diabetes society which is in accordance to the international diagnostic criteria (Fasting Glucose in blood plasma > 7,0 mmol/l) and a HbA1c > 7.0%. After an initial letter inviting eligible participants to the study the patients are approached during usual care visits for informed consent. All patients will be contacted in consecutive order of appearance in the practice, regardless the reason for the current encounter.

### Patient exclusion criteria

1. Patients who are not able to read and understand the patient information form due to dementia, illiteracy or insufficient German language skills

2. Patients who contacted the practice for emergencies only or as a substitute practice

3. Patients with oncological diseases and/or an estimated life expectancy of less than six months due to severe diseases

### Data collection

Patients will receive detailed written information on the aim of the study. After giving their written informed consent they will receive the questionnaire, containing the SF-36 [33], the PACIC 5A [35,36] and the PHQ-9 [42], and a stamped envelope with the postal address of the university. The patients are asked to return this questionnaire in the envelope to the university. Patients will also be informed that neither the GP nor the practice team has any possibility to get knowledge of their answers. The GP will create a list with the participants and allocate a code to each patient. This code is also marked on the questionnaires. The university only receives the patients' codes and has no access to their names. A second questionnaire is filled out by the GP and the practice nurse for each participant regarding current diagnostic findings (e.g. HbA1c, BD, LDL-cholesterol, weight, pulse etc.), comorbidities, medication, diabetes associated complications, compliance, smoking status and quality indicators for diabetes. This questionnaire is marked as well with the patient's code and will be returned to the university in a stamped envelope.

An independent research assistant of the university will enter the data directly into the SPSS program (version 18.0 or higher). Measurements and analysis will take place before randomization (pre-data-collection) and after 12 months (post-data-collection). Pre-data-collection is estimated to last 3 months (months 2 - 4).

### Analysis

We aim to show the reduced level of HbA1c (primary endpoint) in the intervention group using a t-test for independent groups comparison. For binary outcome measures such as success meeting HbA1c, BP and LDL goals, logistic regression will be used to model the relationships between the outcome and treatment group, age and gender. Other potentially important covariates will be identified through exploratory analyses. The longitudinal aspect of the data can be incorporated into the model in various ways, we will utilize the model generalized estimating equations (GEE) approach [52].

For continuous outcomes such as HbA1c levels, blood pressure, lipid levels, SF-36 and the PACIC 5A a repeated measures analysis of variance is appropriate [53]. Fixed effect parameters will include treatment group, age, gender, and other potentially important covariates as the PHQ-9 score.

The primary data analysis will follow the intent-to-treat (ITT) approach where appropriate. This means that all available data from all individuals will be analyzed according to treatment group assignment, regardless of whether or not each individual actually received the assigned treatment.

### Intervention

#### 1. Intervention on the provider of health care

Implementation of complex interventions in a primary care setting is challenging [54]. Our intervention aims at providing team care according to the CCM. To perform care according to the CCM, a team approach involving the practice nurse is required. This represents a further challenge since practice nurses are currently only marginally involved in the care for patients. Thus, we performed a qualitative pre-study with the aim to assess:

1. To what extent the CCM or its elements can be implemented in general practice in Switzerland [55], and

2. How, and to what extent the practice nurse can be involved. The interventions reported below (see "clinical interventions") are carefully adapted to the results of this study [55].

So, our intervention aims at GPs as well as at practice nurses. Both are informed about the intervention of the other group to ease the team approach.

##### Intervention on the practice nurse

In Switzerland, as in most European countries, the education of practice nurses differs tremendously from the U.S. and it is completely unknown if approaches as in the U.S. can be implemented. The education of practice nurses in Switzerland is less focused on medical issues and addresses mainly administrative matters. Lacking medical knowledge has been identified as an important barrier towards an increased involvement of practice nurse in medical care. Unfortunately, professions as advanced nurse practitioners or physicians' assistants do not or only marginally exist. Therefore, practice nurses represent the only resource for a team approach in primary care. This study challenges the hypothesis that CCM appropriate care can be implemented in primary care. To qualify practice nurses to do so, practice nurses from the intervention group will participate in the well established educational course "Treatment of long term patients - module diabetes" ("Betreuung von Langzeitpatienten - Modul Diabetes"). The course lasts 6 days and will be organized by the union of Swiss practice nurses ("Schweizerischer Verband medizinischer Praxisassistentinnen") [56]. The content of the course is the treatment of diabetes patients (medical basics, diet, practical tips, communication etc.), the role of the practice nurse in a team providing structured care for chronically ills and how to perform a follow-up with the CARAT-monitoring-tool. The tool addresses clinical parameters (e.g. HbA1c, blood pressure, LDL etc.), performed examinations (food control, neurological tests, eye examination etc.) as well as referrals and adherence to the prescribed drugs and other recommendations. The tool contains a traffic-light-scheme, indicating if worsening or improvement has occurred. While giving a quick overlook over the status, the traffic-light-scheme will ease the follow-up for the physician who receives the CARAT-tool after it has been filled out by the practice nurse.

##### Intervention on the GPs

The implementation strategy consists of two interactive workshops of 4 hours each including all GPs of the intervention group. The first meeting will take place immediately after randomization of the practices, the second one is intended to have some "booster"-effect and therefore will take place after 3-4 months. In both meetings, the second two hours will contain the GPs as well as the practice nurses to enhance the team approach. The educational objectives of the two meetings are evidence-based treatment of diabetes mellitus and cardiovascular risk factor management in a primary care setting, improvement of the team approach in the practice with focus on the implementation of the CCM elements and knowledge of the Assessment of Chronic Illness Care (ACIC) tool including assessment of the current state in the own practice. The workshops will be conducted with expert presentations and guided discussions together with a GP and a practice nurse who are already experienced in the team approach. Additionally, GPs will receive a written summary of the concept of the CCM as well as information on evidence-based treatments of diabetes in a primary care setting.

#### 2. Clinical intervention - Intervention on the patient

The Chronic Care Model has been shown to provide interactions between patients and providers that support optimal patient functional and clinical outcomes [57,58]. Practice nurses were chosen to implement and to deliver the intervention because of the ongoing need for assessment of goal attainment and because of their ability to work with patients to reduce ambivalence to behavior change, collaborate with GPs and reinforce diabetes education. In the European setting, external nurse practitioners do not exist or are not accepted by GPs. In contrast, the practice nurses are already integrated in the primary care setting and have a continuous relationship with study participants including both direct clinical interventions and collaboration with their GP.

The 6 key elements of the CCM will be addressed as follows:

Organization of health care: The specially trained practice nurse will be involved in care. She will monitor the patient during the study with the CARAT-tool. Contacts for monitoring are planned at least every 4 moths but the frequency can be increased according to the clinical situation of the patient. The clinical aim is to assure that standing orders for established clinical practice guidelines regarding frequency of laboratory testing (HbA1c, LDL, nephropathy screening), yearly ophthalmologic exam and performance of foot exam by the nurses facilitates are met.

Clinical information systems: The information on monitoring and appointments with the practice nurse will be collected in the CARAT-monitoring-tool and will be forwarded to the GP based on importance/urgency before the patient encounter to the GP. This way of information transfer between practice nurse and GP is new. This improvement in communication should help the GP to get an immediate overview on the current situation of the patient. It should also help to address current problems - as for instance a detected depression - more accurately. Data entry by the nurses into the registry allows the GP to obtain an overview on self-care goals, clinical parameters over time (BP, HbA1C, LDL, last ophthalmologic exam, aspirin use, foot exam) via a single sheet prior to each patient visit, and provides prompts for issues to be addressed at a given visit.

For decision support, evidence-based information on diabetes care and patient information leaflets will be provided to the GPs during the interactive workshops. The practice nurses collaborate with the GP by sharing clinical and/or management issues and by providing guideline recommendations for diabetes [1] and depression [59], thus supporting the physician in appropriate decisions. Furthermore, a diabetes specialist of the department of endocrinology at the University Hospital of Zurich will be available for GPs to discuss urgent questions regarding the treatment, to facilitate referrals to specialized care and to make referrals more appropriate.

Self-management support, another key element of the CCM, will be addressed by the practice nurses in the follow-up appointments: Basic skills such as glucose monitoring, insulin administration, teaching about diabetes and its complications, and features of medical nutrition therapy are taught to the study participants with an emphasis on the patient's own life priorities and internal motivations. The aim is to assist the patient in selecting appropriate, concrete behavioral goals, in developing plans for reaching those goals and in evaluating the progress and adequacy of those plans. Specific behavior goals are based on ADA Clinical Guidelines [19] and include (a) dietary adherence, (b) moderate exercise 30 min for 3 days/week, adjusted for patient ability, (c) medication adherence and (d) monitoring.

To inform the patient about community resources, information leaflets will be provided by the practice nurses.

Individualized patient follow-up: Nurse case managers meet individually with patients in the treatment group throughout the study. On average, one hour is spent in each patient visit, with telephone and e-mail correspondence supplementing office visits where appropriate. Follow-up visits include reinforcement of behavior change goals, clinical assessments and attainment of clinical goals.

### Timeframe of the study

The recruitment of the practices will start in December 2009. The patient inclusion will take part in the first quarter of 2010. The interventions/courses will start in April 2010. Assessments will be made as described above, T1 measurement will be performed one year after the T0.

### Description of risks

Serious risks or undesired effects of the CCM or the assessment by questionnaires have not been described in the literature. The clinical diabetes therapy will be oriented according to the available evidence. There are no specific risks related to the study.

### Ethical principles

The study is being conducted in accordance with medical professional codex and the Helsinki Declaration as of 1996 as well as Data Security Laws.

Study participation of patients is voluntary and can be cancelled at any time without provision of reasons and without negative consequences for their future medical care.

### Patient informed consent

Previous to study participation patients receive written and spoken information about the content and extent of the planned study; for instance about potential benefits for their health and potential risks. In case of acceptance they sign the informed consent form.

In case of study discontinuation all material will be destroyed or the patient will be asked if he/she accepts that existing material can be analyzed in the study.

### Vote of the ethics committee

The study protocol has been approved by the ethics committee of the Kanton Zurich and received an unrestricted positive vote on 25.01.2010.

### Data security/disclosure of original documents

The patient names and all other confidential information fall under medical confidentiality rules and are treated according to appropriate Federal Data Security Laws. The results of the patient questionnaires are not accessible to the GPs. Questionnaires are directly mailed to the study centre by the patient.

All study related data and documents are stored on a protected central server of the University of Zurich. Only direct members of the internal study team can access the respective files.

Intermediate and final reports are stored in the office of the Department of General Practice and Health Services Research at the Zurich University Hospital (USZ).

## Discussion

Chronic conditions and multimorbidity represent the major challenge for the health care systems in the industrialized world. The CCM provides a proactive, patient-centred, evidence-based approach to face this challenge. In many recent studies, positive results on patient relevant outcomes could be shown. Adam et al. showed in a metaanalyses that patients with asthma, receiving care that included at least two elements of the CCM, were less often hospitalized and had less emergency encounters [60]. Nutting et al. showed that the implementation of CCM elements could be performed in single handed primary care practices without major efforts or structural changes. This implementation improved clinical parameters as well as process parameters [61]. In another study, Parchman et al. showed that the extent to which care complies with the CCM is a predictor for coronary vessel disease in a 10 year time frame [62]. Vargas et al. showed that the care according to the CCM is able to improve risk factors for cardiovascular diseases in a sample of patients with diabetes [63]. Based on the growing evidence that the CCM or at least some of its elements have positive effects on patients' health, the recent literature contains recommendations to arrange care according to the CCM for CVD patients [64], diabetics [62] but also patients with depression [65].

Taking into account the increasing evidence regarding the effects of the CCM, the German Society for General Practice and Family Medicine (Deutsche Gesellschaft für Allgemein- und Famillienmedizin; DEGAM) has launched a position paper during its annual meeting in autumn 2006. In this statement, the CCM has explicitly been suggested as template for the care for chronically ills in Germany [7,8]. But even the evidence for the CCM is growing worldwide, it has to be acknowledged that these data haven been retrieved in health care systems which differ in many aspects from the German health care system. The resources regarding medical professionals as nurse practitioners are completely different in Switzerland compared to the U.S., for example. But these professionals play an important role in the CCM. So far, no experiences are available with implementation of the CCM in the Swiss health care system. Furthermore, the CCM has been evaluated regarding diabetes in HMOs, data from small, singled handed primary care practices are not available.

Therefore, this study will assess if the CCM can be implemented in the Swiss health care system as well as if the CCM can improve the diabetes treatment. Positive results could not only impact current diabetes treatment but also enhance the discussed team orientation in primary care.

The primary goal of the CARAT trial is to evaluate the effectiveness of an enhanced case management provided by the practice nurse to improve clinical and psychological outcomes in patients with diabetes in primary care setting, thus determining the impact and sustainability of the intervention on glycemic and lipid control over 1 year. The intervention is designed to incorporate aspects of the CCM with the addition of self-management support through education. In addition, extensive nurse training and increased attention to clinical care guidelines will improve outcomes. The study addresses important questions regarding the use of nurse case managers in overall diabetes care, the role of health care providers to initiate or intensify therapy when indicated, and the psychosocial effects of diabetes on emotional distress, quality of life and self-care behaviors.

## Limitations

It should be acknowledged that the sample of GPs is associated with some kind of selection bias because only GPs who are interested in the topic might have intended the information meeting. Such a selection bias can not be avoided since participation is based on GPs free choice. It might also not limit the implications of an assumed positive result for daily practice: If a significant difference could be achieved in this group of GPs who might be more familiar with or/and interested in diabetes treatment it could be assumed that this effect could also be observed if the intervention is implemented in regular, weaker performing GPs practices. The threshold for significance is higher since we are comparing the intervention group with an assumable already well performing control group.

## List of abbreviations

Abbreviations: BP: blood pressure; LDL: low-density lipoprotein; GP: general practitioner; RCT: randomized-controlled trial; QoL: quality of life.

## Competing interests

The authors declare that they have no competing interests.

## Authors' contributions

TR, HS and BB were the initiators for this study, TR drafted the study protocol. AF and TR organized the recruitment of the practices. AF, CC and TR developed the questionnaires, organized the data collection and management and the administration of the whole study. CC and TR developed the CARAT-Monitoring-Tool. TR, UH and JS conducted the power calculation, supervised the analyses and gave statistical and methodical input. BB is the organizer of the practice nurses' educational course, TR, AF, CC, HS and BB will organize the interactive workshops. TR, AF and CC wrote and revised the final manuscript, and all authors read and approved it.

## References

[B1] World Health Organization The World Health Report 2003: Shaping the future 2003

[B2] Epping-Jordan JE Pruitt SD Bengoa R Wagner EH Improving the quality of health care for chronic conditions Qual Saf Health Care 2004 13 4 299 305 10.1136/qshc.2004.010744 15289634 PMC1743863

[B3] Tsai AC Morton SC Mangione CM Keeler EB A meta-analysis of interventions to improve care for chronic illnesses Am J Manag Care 2005 11 8 478 488 16095434 PMC3244301

[B4] Wagner EH Chronic disease management: what will it take to improve care for chronic illness? Eff Clin Pract 1998 1 1 2 4 10345255

[B5] Wagner EH Austin BT Davis C Hindmarsh M Schaefer J Bonomi A Improving chronic illness care: translating evidence into action Health Aff (Millwood) 2001 20 6 64 78 10.1377/hlthaff.20.6.64 11816692

[B6] Lenfant C Shattuck lecture--clinical research to clinical practice--lost in translation? N Engl J Med 2003 349 9 868 874 10.1056/NEJMsa035507 12944573

[B7] Gensichen J Muth C Butzlaff M Rosemann T Raspe H de Cornejo GM Beyer M Harter M Muller UA Angermann CE [The future is chronic: German primary care and the Chronic Care Model--The comprehensive principles in the proactive treatment of the chronically ill] Z Arztl Fortbild Qualitatssich 2006 100 5 365 374 16955622

[B8] Gerlach FM Beyer M Saal K Peitz M Gensichen J [New perspectives in the primary care of the chronically ill--against the "tyranny of the urgent". Part 2: The chronic care model und case management as the basis of a forward-looking approach to primary care] Z Arztl Fortbild Qualitatssich 2006 100 5 345 352 16955620

[B9] Heidemann C Kroll L Icks A Lampert T Scheidt-Nave C Prevalence of known diabetes in German adults aged 25-69 years: results from national health surveys over 15 years Diabet Med 2009 26 6 655 658 10.1111/j.1464-5491.2009.02739.x 19538243

[B10] Prevention CfDCa National diabetes fact sheet: general information and national estimates on diabetes in the United States 2005

[B11] American Diabetes Association Economic costs of diabetes in the US in 2007 Diabetes care 2008 31 3 1 20 10.2337/dc08-9017 18308683

[B12] Bouma M Dekker JH van Eijk JT Schellevis FG Kriegsman DM Heine RJ Metabolic control and morbidity of type 2 diabetic patients in a general practice network Fam Pract 1999 16 4 402 406 10.1093/fampra/16.4.402 10493712

[B13] Szecsenyi J Rosemann T Joos S Peters-Klimm F Miksch A German diabetes disease management programs are appropriate for restructuring care according to the chronic care model: an evaluation with the patient assessment of chronic illness care instrument Diabetes care 2008 31 6 1150 1154 10.2337/dc07-2104 18299443

[B14] Bero LA Grilli R Grimshaw JM Harvey E Oxman AD Thomson MA Closing the gap between research and practice: an overview of systematic reviews of interventions to promote the implementation of research findings. The Cochrane Effective Practice and Organization of Care Review Group Bmj 1998 317 7156 465 468 9703533 10.1136/bmj.317.7156.465 PMC1113716

[B15] Thomson MA Oxman AD Davis DA Haynes RB Freemantle N Harvey EL Audit and feedback:effects on professional practice and health care outcomes (Cochrane Review) The Cochrane Library 2001 1 10.1002/14651858.CD000259 10796520

[B16] Wensing M van der Weijden T Grol R Implementing guidelines and innovations in general practice: which interventions are effective? Br J Gen Pract 1998 48 427 991 997 9624774 PMC1409988

[B17] Thomson MA Oxman AD Davis DA Haynes RB Freemantle N Harvey EL Educational outreach visits: effects on professional practice and health care outcomes. (Cochrane Review) The Cochrane Library 2001 1 10.1002/14651858.CD000409 10796542

[B18] von Ferber L Koster I Hauner H Medical costs of diabetic complications total costs and excess costs by age and type of treatment results of the German CoDiM Study Exp Clin Endocrinol Diabetes 2007 115 2 97 104 10.1055/s-2007-949152 17318768

[B19] American Diabetes Association Clinical practice recommendations Diabetes care 2008 31 1 1 110 10.2337/dc08-S001

[B20] Anderson RM Funnell MM The art of empowerment: psychology in diabetes care 2000

[B21] Rubin RR Peyrot M Quality of life and diabetes Diabetes Metab Res Rev 1999 15 3 205 218 10.1002/(SICI)1520-7560(199905/06)15:3<205::AID-DMRR29>3.0.CO;2-O 10441043

[B22] Lustman PJ Griffith LS Clouse RE Depression in adults with diabetes. Results of 5-yr follow-up study Diabetes care 1988 11 8 605 612 10.2337/diacare.11.8.605 3219966

[B23] de Groot M Jacobson AM Samson JA Welch G Glycemic control and major depression in patients with type 1 and type 2 diabetes mellitus J Psychosom Res 1999 46 5 425 435 10.1016/S0022-3999(99)00014-8 10404477

[B24] Anderson RJ Freedland KE Clouse RE Lustman PJ The prevalence of comorbid depression in adults with diabetes: a meta-analysis Diabetes care 2001 24 6 1069 1078 10.2337/diacare.24.6.1069 11375373

[B25] Goldstein BJ Muller-Wieland D Textbook of type 2 diabetes 2003

[B26] Lustman PJ Clouse RE Treatment of depression in diabetes: impact on mood and medical outcome J Psychosom Res 2002 53 4 917 924 10.1016/S0022-3999(02)00416-6 12377304

[B27] Lustman PJ Griffith LS Freedland KE Kissel SS Clouse RE Cognitive behavior therapy for depression in type 2 diabetes mellitus. A randomized, controlled trial Ann Intern Med 1998 129 8 613 621 9786808 10.7326/0003-4819-129-8-199810150-00005

[B28] Resnick HE Foster GL Bardsley J Ratner RE Achievement of American Diabetes Association clinical practice recommendations among U.S. adults with diabetes, 1999-2002: the National Health and Nutrition Examination Survey Diabetes care 2006 29 3 531 537 10.2337/diacare.29.03.06.dc05-1254 16505501

[B29] Saydah SH Fradkin J Cowie CC Poor control of risk factors for vascular disease among adults with previously diagnosed diabetes Jama 2004 291 3 335 342 10.1001/jama.291.3.335 14734596

[B30] Schmittdiel JA Uratsu CS Karter AJ Heisler M Subramanian U Mangione CM Selby JV Why don't diabetes patients achieve recommended risk factor targets? Poor adherence versus lack of treatment intensification Journal of general internal medicine 2008 23 5 588 594 10.1007/s11606-008-0554-8 18317847 PMC2324158

[B31] Saaddine JB Cadwell B Gregg EW Engelgau MM Vinicor F Imperatore G Narayan KM Improvements in diabetes processes of care and intermediate outcomes: United States, 1988-2002 Ann Intern Med 2006 144 7 465 474 16585660 10.7326/0003-4819-144-7-200604040-00005

[B32] Buse JB Ginsberg HN Bakris GL Clark NG Costa F Eckel R Fonseca V Gerstein HC Grundy S Nesto RW Primary prevention of cardiovascular diseases in people with diabetes mellitus: a scientific statement from the American Heart Association and the American Diabetes Association Circulation 2007 115 1 114 126 10.1161/CIRCULATIONAHA.106.179294 17192512

[B33] Bullinger M Kirchberger I Ware J Der deutsche SF-36 Health Survey. Übersetzung und psychometrische Testung eines krankheitsübergreifenden Instruments zur Erfassung der gesundheitsbezogenen Lebensqualität Zeitschrift für Gesundheitswissenschaften 1995 3 21 36

[B34] American Diabetes Association Standards of Medical Care in Diabetes - 2010 Diabetes care 2010 33 Supplement 1 S11 S61 10.2337/dc10-S011 20042772 PMC2797382

[B35] Glasgow RE Wagner EH Schaefer J Mahoney LD Reid RJ Greene SM Development and validation of the Patient Assessment of Chronic Illness Care (PACIC) Med Care 2005 43 5 436 444 10.1097/01.mlr.0000160375.47920.8c 15838407

[B36] Rosemann T Laux G Droesemeyer S Gensichen J Szecsenyi J Evaluation of a culturally adapted German version of the Patient Assessment of Chronic Illness Care (PACIC 5A) questionnaire in a sample of osteoarthritis patients J Eval Clin Pract 2007 13 5 806 813 10.1111/j.1365-2753.2007.00786.x 17824876

[B37] Goldstein MG Whitlock EP DePue J Multiple behavioral risk factor interventions in primary care. Summary of research evidence Am J Prev Med 2004 27 2 Suppl 61 79 10.1016/j.amepre.2004.04.023 15275675

[B38] Glasgow RE Whitesides H Nelson CC King DK Use of the Patient Assessment of Chronic Illness Care (PACIC) with diabetic patients: relationship to patient characteristics, receipt of care, and self-management Diabetes care 2005 28 11 2655 2661 10.2337/diacare.28.11.2655 16249535

[B39] Bonomi AE Wagner EH Glasgow RE VonKorff M Assessment of chronic illness care (ACIC): a practical tool to measure quality improvement Health ServRes 2002 37 3 791 820 10.1111/1475-6773.00049 PMC1434662 12132606

[B40] Lesperance F Frasure-Smith N Talajic M Major depression before and after myocardial infarction: its nature and consequences Psychosom Med 1996 58 2 99 110 8849624 10.1097/00006842-199603000-00001

[B41] Morris P Robinson R Andrzejewski P Samuels J Price T Association of depression with 10-year poststroke mortality Am J Psychiatry 1993 150 1 124 129 8417554 10.1176/ajp.150.1.124

[B42] Lowe B Kroenke K Herzog W Grafe K Measuring depression outcome with a brief self-report instrument: sensitivity to change of the Patient Health Questionnaire (PHQ-9) J Affect Disord 2004 81 1 61 66 10.1016/S0165-0327(03)00198-8 15183601

[B43] Gensichen J Torge M Peitz M Wendt-Hermainski H Beyer M Rosemann T Krauth C Raspe H Aldenhoff JB Gerlach FM Case management for the treatment of patients with major depression in general practices--rationale, design and conduct of a cluster randomized controlled trial--PRoMPT (PRimary care Monitoring for depressive Patient's Trial) [ISRCTN66386086]--study protocol BMCPublic Health 2005 5 101 10.1186/1471-2458-5-101 PMC1262729 16207375

[B44] Rosemann T Joos S Laux G Gensichen J Szecsenyi J Case management of arthritis patients in primary care: a cluster-randomized controlled trial Arthritis and rheumatism 2007 57 8 1390 1397 10.1002/art.23080 18050178

[B45] Campbell MK Mollison J Grimshaw JM Cluster trials in implementation research: estimation of intracluster correlation coefficients and sample size Stat Med 2001 20 3 391 399 10.1002/1097-0258(20010215)20:3<391::AID-SIM800>3.0.CO;2-Z 11180309

[B46] Campbell M Grimshaw J Steen N Sample size calculations for cluster randomised trials. Changing Professional Practice in Europe Group (EU BIOMED II Concerted Action) J Health Serv Res Policy 2000 5 1 12 16 10787581 10.1177/135581960000500105

[B47] Piccinelli M Wilkinson G Gender differences in depression. Critical review Br J Psychiatry 2000 177 486 492 10.1192/bjp.177.6.486 11102321

[B48] University of Aberdeen Empirical estimates of ICCs from changing professional practice studies 2005

[B49] Ott P Benke I Stelzer J Köhler C Hanefeld M „Diabetes in Germany" (DIG)-Studie Deutsche medizinische Wochenschrift 2009 134 07 291 297 10.1055/s-0028-1123994 19197810

[B50] The Action to Control Cardiovascular Risk in Diabetes Study Group Effects of Intensive Glucose Lowering in Type 2 Diabetes N Engl J Med 2008 358 24 2545 2559 10.1056/NEJMoa0802743 18539917 PMC4551392

[B51] Campbell MK Thomson S Ramsay CR MacLennan GS Grimshaw JM Sample size calculator for cluster randomized trials ComputBiolMed 2004 34 2 113 125 10.1016/S0010-4825(03)00039-8 14972631

[B52] Diggle PJPH Liang K-Y Zeger SL Analysis of longitudinal data 2002 2

[B53] Vonesh EFVC Linear and nonlinear models for the analysis of repeated measurements 1997

[B54] Campbell MJ Cluster randomized trials in general (family) practice research Stat Methods Med Res 2000 9 2 81 94 10.1191/096228000676246354 10946428

[B55] Rosemann T Joest K Korner T Schaefert R Heiderhoff M Szecsenyi J How can the practice nurse be more involved in the care of the chronically ill? The perspectives of GPs, patients and practice nurses BMC Fam Pract 2006 7 14 10.1186/1471-2296-7-14 16515692 PMC1475585

[B56] Schwiezerischer Verband Medizinischer PraxisAssiistentinnen http://www.sva.ch/no_cache/bildung/weiterbildung/weiterbildung-angebote/weiterbildung-detailansicht/article/betreuung-von-langzeitpatienten-modell-diabetes.html?tx_ttnews%5BbackPid%5D=181

[B57] Bodenheimer T Wagner EH Grumbach K Improving primary care for patients with chronic illness Jama 2002 288 14 1775 1779 10.1001/jama.288.14.1775 12365965

[B58] Bodenheimer T Wagner EH Grumbach K Improving primary care for patients with chronic illness: the chronic care model, Part 2 Jama 2002 288 15 1909 1914 10.1001/jama.288.15.1909 12377092

[B59] Barry SL Oxman TE Care manager training module: three component model for management of depression 200415335127

[B60] Adams SG Smith PK Allan PF Anzueto A Pugh JA Cornell JE Systematic review of the chronic care model in chronic obstructive pulmonary disease prevention and management Archives of internal medicine 2007 167 6 551 561 10.1001/archinte.167.6.551 17389286

[B61] Nutting PA Dickinson WP Dickinson LM Nelson CC King DK Crabtree BF Glasgow RE Use of chronic care model elements is associated with higher-quality care for diabetes Annals of family medicine 2007 5 1 14 20 10.1370/afm.610 17261860 PMC1783920

[B62] Parchman ML Pugh JA Wang CP Romero RL Glucose control, self-care behaviors, and the presence of the chronic care model in primary care clinics Diabetes care 2007 30 11 2849 2854 10.2337/dc06-2516 17682121

[B63] Vargas RB Mangione CM Asch S Keesey J Rosen M Schonlau M Keeler EB Can a chronic care model collaborative reduce heart disease risk in patients with diabetes? Journal of general internal medicine 2007 22 2 215 222 10.1007/s11606-006-0072-5 17356989 PMC1824758

[B64] Block RC Pearson TA Organizing services for cardiovascular prevention Current treatment options in cardiovascular medicine 2007 9 4 278 286 10.1007/s11936-007-0023-4 17761113

[B65] McEvoy P Barnes P Using the chronic care model to tackle depression among older adults who have long-term physical conditions Journal of psychiatric and mental health nursing 2007 14 3 233 238 10.1111/j.1365-2850.2007.01066.x 17430445

